# Large plasmids encoding antibiotic resistance and localized-like adherence in atypical enteropathogenic *Escherichia coli* strains

**DOI:** 10.1186/s12866-020-01809-4

**Published:** 2020-05-29

**Authors:** Scarlat S. Silva, Mariane V. Monfardini, Isabel C. A. Scaletsky

**Affiliations:** Departamento de Microbiologia, Imunologia e Parasitologia, Universidade Federal de São Paulo, Escola Paulista de Medicina, Rua Botucatu, 862, 3 andar, São Paulo, 04023-062 Brazil

**Keywords:** Atypical enteropathogenic *Escherichia coli*, Localized adherence-like, Antimicrobial resistance, Plasmids

## Abstract

**Background:**

In previous studies, we have shown that atypical enteropathogenic *Escherichia coli* (aEPEC) strains are important diarrheal pathogens among Brazilian children. In the characterization of a collection of 126 aEPEC strains, we identified 29 strains expressing the localized-like adherence (LAL) pattern on HEp-2 cells and harboring large plasmids in the range of 60 to 98 MDa. In this study, we examined 18 of these strains for their ability to transfer the LAL phenotype to a *E. coli* K-12 C600 strain.

**Results:**

In conjugation experiments, using eight strains which were resistant to one or more antimicrobials and positive for F-pili genes (*traA*), we were able to cotransfer antimicrobial resistance markers along with adhesion genes. By transforming *E. coli* DH5α with plasmid DNA from strains A46 (pIS46), A66 (pIS66) and A102 (pIS102), we were able to demonstrate that genes encoding ampicillin, tetracycline and LAL were encoded on a 98-MDa conjugative plasmid. To identify a gene responsible for LAL, we constructed a transposon mutant library of A102 strain. Among 18 mutants that did not adhere to HeLa cells, four carried insertions within fimbrial genes (*fimA* and *traJ)* and agglutinin genes *(tia* and *hek)*. Using these Tn5 mutants as donors, we were able to obtain kanamycin-resistant *E. coli* MA3456 transconjugants. Sequence analysis of the plasmid genes revealed a region exhibit to 80 and 73% amino acid similarities to the agglutinins Tia and Hek, respectively.

**Conclusion:**

In this study, we have identified three large conjugative plasmids, pIS46, pIS66 and pIS102, coding for antimicrobial resistance and localized-like adherence (LAL) to HeLa cells. In addition, we identified a *tia*/*hek* homolog encoded on the pIS102 plasmid, which seems to be involved in adhesion of A102 strain.

## Background

Enteropathogenic *Escherichia coli* (EPEC) is a leading cause of infantile diarrhea in developing countries, including Brazil [1–3]. EPEC colonizes the small intestine, causing characteristic attaching and effacing (A/E) lesions in the intestinal epithelial cells. The genes necessary for the A/E phenotype are located on a pathogenicity island named the locus of enterocyte effacement (LEE), which encodes a type III secretion system and effectors, the outer membrane adhesin intimin and its translocated receptor (Tir) [4–6].

EPEC is divided into typical (tEPEC) and atypical (aEPEC) strains [7, 8]. Typical EPEC strains carry a large virulence plasmid designated the EPEC adherence factor plasmid (pEAF) [9]. The pEAF plasmid encodes the bundle forming pilus (Bfp), which promote bacterium-to-bacterium adherence, resulting in formation of compact microcolonies on the surface of HeLa/HEp-2 cells after 3 h of incubation, a phenotype known as localized adherence (LA) [10, 11]. In contrast, atypical EPEC strains do not posses the pEAF plasmid, and are unable to produce LA. In the absence of Bfp, atypical EPEC strains display a variant LA pattern designated LA-like (LAL) pattern, which is characterized by the presence of compact microcolonies on HEp-2 cells observed after 6 h of infection [12]. LAL is the most common pattern seen among aEPEC strains, however, some strains exhibit diffuse adherence (DA) or aggregative adherence (AA) patterns [13–15]. However, not much is known about the adherence factors involved with these phenotypes. In a previous study, we identified two factors contributing to the LAL phenotype of an aEPEC strain *E. coli* 22 (O26:NM) [16]. A novel afimbrial adhesin called the locus for diffuse adherence, *lda*, which encodes a diffuse pattern of adherence on HEp-2 cells when cloned into a non-adherent *E. coli* K12 strain. A second plasmid-encoded factor that contributes to the compact microcolony formation of *E. coli 22*, but was not characterized.

Atypical EPEC is currently an emerging diarrheal pathogen in both developing and developed countries [17–20]. In previous studies, we have shown that classic aEPEC strains are important diarrheal pathogens among Brazilian children [21, 22]. In the characterization of a collection of 126 aEPEC strains, we identified 29 strains expressing the LAL pattern on HEp-2 cells [23]. Most of these strains belonged to the classical EPEC serotypes and carried one or two large plasmid bands in the range of 60 to 98 MDa. In this study, we sought to investigate whether these plasmids are involved in the LAL phenotype.

## Results

### Examination of plasmids in aEPEC LAL^+^

In this study, we examined 18 aEPEC LAL^+^ strains harboring large plasmids and belonging to different serotypes for their ability to transfer the LAL phenotype to a *E. coli* K-12 strain. Strains were characterized for antibiotic resistance to ampicillin (Ap), tetracycline (Tc), chloramphenicol (Cm), kanamycin (Km), and nalidixic acid (Nal), and screened for the presence of conjugal transfer (*tra*) genes. Among the 18 LAL^+^ aEPEC strains, 12 were resistant to one or more antimicrobials, and eight of these were positive for F-pili genes (*traA*) (Table 1).

**Table 1:** Results of conjugation between LAL^+^ aEPEC strains and *E. coli* K-12 C600

aEPEC strain	Source	Serotype	Resistance profile^a^	Size (MDa) of plasmid(s)^b^	Presence (+) or absence (−) of traA	Resistance profile of transconjugant(s)	Size (MDa) of transconjugant plasmid(s)	LAL phenotype
A1	Diarrhea	O26:NM	–	~ 60, 4.6	–	–	–	–
A24	Diarrhea	O26:NM	Ap^r^	98	+	Ap^r^ Nal^r^	98	–
A14	Diarrhea	O26:HND	Km^r^ Nal^r^	98, ~ 60	–	–	–	–
A72	Diarrhea	O26:HND	Km^r^ Nal^r^	~ 60	–	–	–	–
A153	Diarrhea	O26:HND	–	98, < 4.6	+	–	–	–
A46	Diarrhea	O55:HND	Ap^r^ Km^r^ Tc^r^	~ 60, 98	+	Ap^r^ Tc^r^ Nal^r^	98	+
A76	Diarrhea	O85:H40	Ap^r^ Cm^r^	~ 60	+	Ap^r^ Cm^r^ Nal^r^	98	–
A17	Control	O105:H7	–	~ 60	+	–	–	–
A12	Control	O111:NM	Km^r^ Nal^r^	75, 4.6	–	–	–	–
A60	Diarrhea	O119:H2	–	98	+	–	–	–
A66	Diarrhea	O119:HND	Ap^r^ Tc^r^	98	+	Ap^r^ Tc^r^ Nal^r^	98	+
A13	Diarrhea	O126:NM	–	65, < 4.6	–	–	–	–
A11	Diarrhea	O142:NM	–	65, 4.6	+	–	–	–
A104	Diarrhea	ONT:H18	Ap^r^ Tc^r^	> 98	+	Ap^r^ Tc^r^ Nal^r^	> 98	–
A106	Diarrhea	ONT:H18	Ap^r^ Cm^r^ Tc^r^	> 98	+	Ap^r^ Tc^r^ Nal^r^	> 98	–
A10	Diarrhea	ONT:HND	–	> 98	+	–	–	–
A102	Diarrhea	ONT:HND	Ap^r^ Km^r^ Tc^r^	98	+	Ap^r^ Tc^r^ Nal^r^	98	+
A128	Diarrhea	ONT:HND	Ap^r^ Tc^r^	98	+	Ap^r^ Tc^r^ Nal^r^	98	–

^a^Ap^r^, ampicillin resistant; Km^r^, kanamycin resistant; Cm^r^, chloramphenicol resistant; Tc^r^, tetracycline resistant; Nal^r^, nalidicix acid resistant

^b^The size of plasmids was calculated based on relative migration of plasmids with known sizes contained in strain 39-R861 [24]

**Fig. 1:**
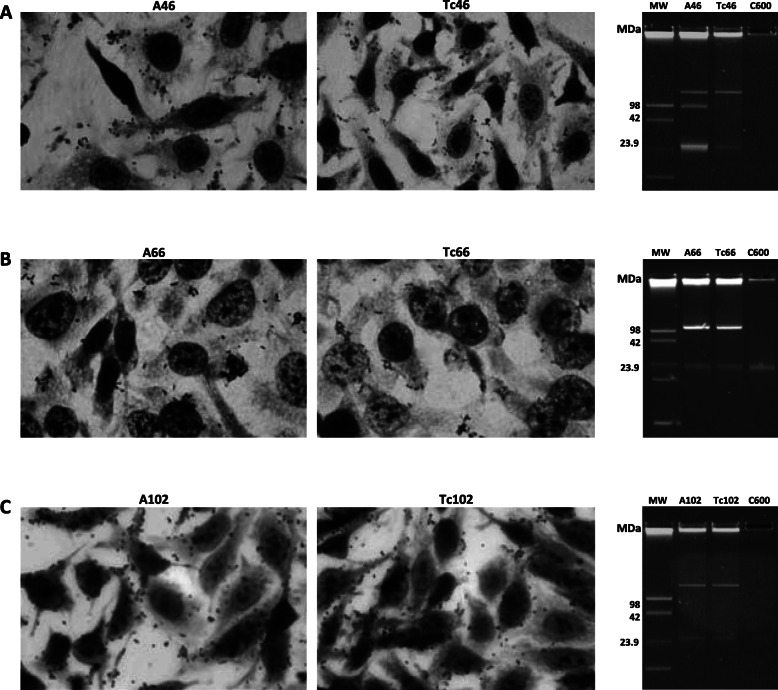
HeLa cell adherence assay and plasmid profiles of the aEPEC and transconjugant strains. Light microscopy micrographs at 3 h after infection showing the localized-like adherence (LAL) (original magnification, 400x). **a** aEPEC A46 and transconjugant, **b** aEPEC A66 and transconjugant, and (**c**) aEPEC A102 and transconjugant. C600, a non-adherent and plasmidless *E. coli* K12 strain. MW, 39-R861strain carrying plasmids of known molecular sizes [24]

To test for the presence of LAL plasmids, we perfomed conjugation experiments with the resistant *traA*-positive strains and the plasmidless non-adherent *E. coli* K-12 C600 strain. As shown in Table 1, all eight *traA*-positive strains transferred multiple antibiotic resistance to *E. coli* C600 strain. The transfer frequencies were low in the range between 10^− 6^ and 10^− 9^. Using two transconjugants of each pattern in a HeLa cell adhesion assay we found that only transconjugants from A46 (O55:HND), A66 (O119:HND) and A102 (ONT:HND) strains exhibited the LAL phenotype (Fig. 1).

**Fig. 2:**
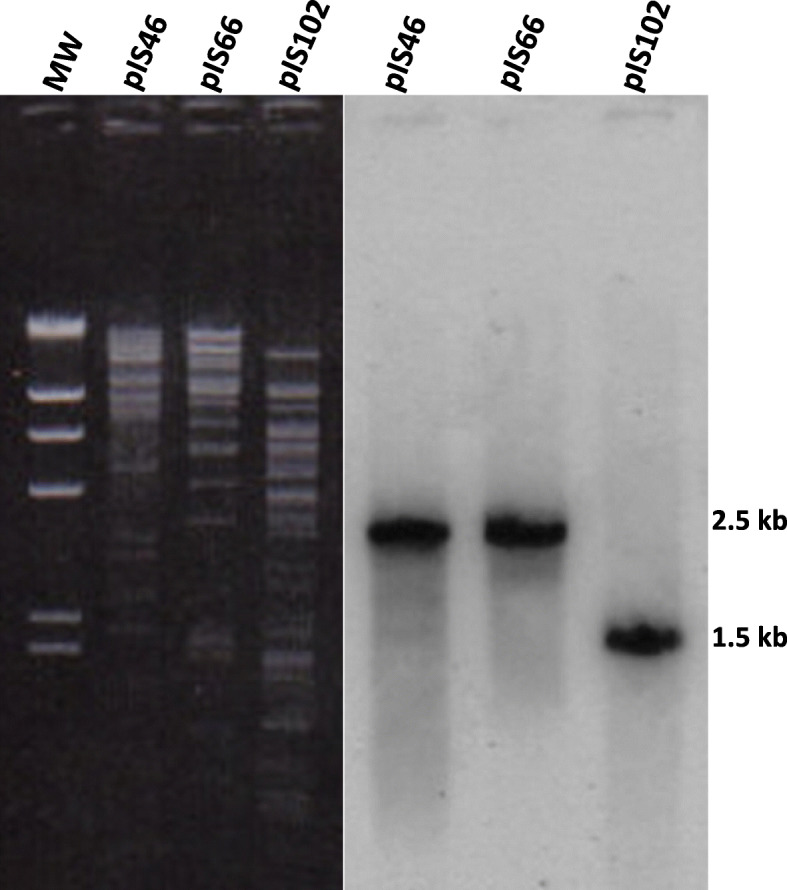
Southern blotting analysis of transconjugant plasmids from strains A46 (pIS46), A66 (pIS66), and A102 (pIS102) after digestion with *Eco*RI using the *traA* gene as a probe. MW, λ/*Hin*dIII molecular marker

Strain A46 carries two plasmids, one of about 60-MDa and another of 98-MDa, being only the larger plasmid the conjugative. The phenotype of donor strain and transconjugant was Ap^r^ Tc^r^ LAL (Fig. 1a). Strain A66 harbors only one large conjugative plasmid of about 98-MDa encoding for Ap^r^ Tc^r^ LAL (Fig. 1b). Strain A102 contain only one large conjugative plasmid of about 98-MDa encoding for Ap^r^ Tc^r^ LAL (Fig. 1c). Southern blot hybridization with *traA* genes detected restriction fragments of 2.5-kb in pIS46 and pIS66 plasmids and 1.4-kb in A102 strain, and revealed different profiles (Fig. 2).

In order to confirm the presence of LAL plasmids, the transconjugant plasmids from strains A46 (pIS46), A66 (pIS66), and A102 (pIS102) were transformed into *E. coli* K-12 DH5α, as described elsewhere [25]. The resulting transformants carrying pIS46, pIS66, or pIS102, were used as donors in a second conjugation experiment with *E. coli* K-12 J53 as the recipient strain. The seletive plates were minimal medium A [26] containing ampicillin and tetracycline. All Ap^r^ Tc^r^ transconjugants showed LAL phenotype.

Nwaneshiudu et al. [27] identified a large conjugative multidrug resistance plasmid in an aEPEC O119:H2 strain MB80. After, we identified variants of the MB80 conjugative resistance in other EPEC strains [28]. In this study, we screened the 18 aEPEC LAL^+^ strains, and all tested negative for the pMB80 *traI* and *traC* PCR*s*.

### Identification of genes involved in LAL phenotype

To identify alternative adhesins to Bfp in aEPEC, we studied strain A102. To identify genes involved in LAL phenotype, we mutagenized strain A102 with the EZ::TN < R6Kγ*ori*/KAN-2> Tnp transposome, and screened for adhesion-defective mutants. Among 1100 transposon-inserted mutants screened, 18 mutants that did not adhere to HeLa cells were isolated. All these 18 mutants showed growth rates comparable to those of the parent strains (data not shown). The transposon-inserted locus of each mutant was cloned, as described in Material and Methods, and insertion flanking regions were sequenced. Fourteen transposon insertions were located in genes associated with amino acid metabolism, outer membrane proteins, transcriptional regulators, DNA enzymes, and transport system. Transposon in four mutants were found within fimbrial genes (*fimA* and *traJ)* and agglutinin genes *(tia* and *hek)* (Table 2).

**Table 2:** Transposon mutagenesis results for strain A102

Mutant strain	Gene function	% amino acid identity
II-H-9	Autotransporter outer membrane beta-barrel domain-containing protein	100%
X-F-12	Nitric oxide reductase [*Escherichia coli*]	100%
III-C-1	Lysine descarboxilase [Escherichia coli B088]	100%
III-A-5	Aspartokinase/Homoserine dehydrogenase [Escherichia coli ISC7]	100%
X-A-12	Oligogalacturonate lyase [Escherichia coli]	100%
I-D-4	GTP-binding protein, partial [Escherichia coli]	100%
III-H-12	Electron transfer flavoprotein, partial [Escherichia coli]	100%
II-E-4	ABC transporter ATP-binding protein [Enterobacteriaceae]	99%
II-E-5	Fimbrial protein [Escherichia coli H120]	99%
V-A-1	DeoR Family transcriptional regulator	99%
IV-B-7	Sodium/proline symporter [Escherichia coli]	99%
II-C-7	draP [Escherichia coli LAU-EC10]	96%
II-E-4	Hypothetical protein, partial	80%
II-A-7	Tia invasion determinant domain protein [Escherichia coli]	80%
II-H-5, X-B-12	ABC transporter ATP-binding protein [Escherichia coli]	75%
II-F-5	Adhesin/Virulence fator Hek [Escherichia coli]	73%
II-D-10	Protein TraJ (Positive regulator of conjugal transfer operon) (Plasmid) [Escherichia coli S88]	73%

**Fig. 3:**
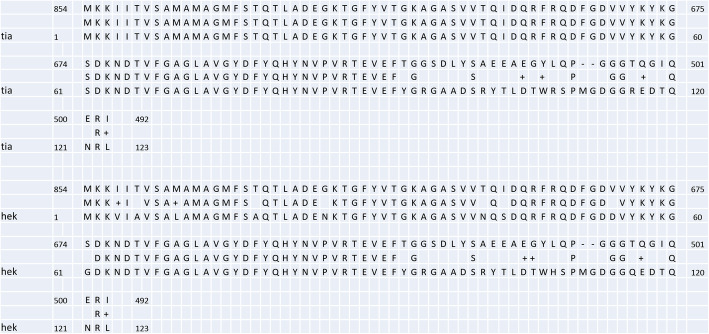
Alignment of the deduced amino acid sequence of the 1175 pb fragment and the tia invasion determinant and the adhesin/virulence factor Hek

In this study, we demonstrated that the pIS102 plasmid confers LAL pattern in HeLa cells when expressed in *E. coli* K-12, and we suspected that the agglutinin genes were located on this plasmid. Thus, we attempted to transfer the agglutinin genes (containing the Tn5-kanamycin) from the mutants II-A-7 and II-F-5 into a nalidixic acid-resistant plasmidless *E. coli* MA3456. Indeed, we were able to obtain kanamycin-resistant *E. coli* MA3456 transconjugants using the Tn5 mutant II-A-7 as donor. Plasmid genes flanking the Tn5-transposon insertion were cloned, and a 1200 bp *Eco*RI fragment containing Tn5 was subjected to DNA sequence. BLAST analysis identified a protein of 123 aminoacids with 80% identity to Tia and 73% identity to Hek (Fig. 3; see Figure S1 in the supplemental material).

## Discussion

The frequency of isolation of aEPEC from diarrheal cases has significantly increased in Brazil in the last years [1–3]. Atypical EPEC strains lack the EAF plasmid and hence are Bfp negative, but they can still adhere to HEp-2 cells in a Bfp independent LA pattern, which is referred to as LAL [12]. The LAL pattern is characterized by the presence of compact microcolonies on HEp-2 cells, but only after 6 h of incubation, whereas LA is apparent after 3 h.

In the search for potential adherence factors, we studied 18 atypical aEPEC LAL^+^ strains isolated in a case control study in Brazil [23]. All of the 18 strains harbor one or two large plasmids and many genes encoding for known *E. coli* adhesins, but to date, no adhesive structure has been implicated in the microcolonies formation of aEPEC [23].

Our results demonstrate a high rate of resistance to certain antimicrobial agents in 12 aEPEC LAL^+^ strains in which resistance is apparently associated with conjugative plasmids. In addition, we also found plasmids encoding multiple drug resistance along with adhesion genes in three aEPEC strains. By transforming *E. coli* DH5α with plasmids from A46, A66 and A102 strains, we were able to demonstrate that genes encoding ampicillin and tetracycline resistance and LAL were encoded on a 98-MDa conjugative plasmid. Although an aEPEC conjugative multiresistance plasmid has been described [27], a plasmid coding for antibiotic resistance along with adhesion genes has not been previously reported in aEPEC.

In this study, we used Tn5-based transposon mutagenesis to identify the genetic determinants of aEPEC A102 strain responsible for the LAL phenotype. We have found that multiple factors, such as amino acid metabolism, transcriptional regulators, or transport systems may affect the adherence of A102 strain to HeLa cells. However, we identified mutated genes associated with fimbrial adhesins (fimA and traJ) and agglutinin genes (tia and hek), which suggest a role of these genes in adherence.

The fimbrial gene *fimA* encodes the larger subunit of type 1 pilus (T1P). T1P has been reported to be responsible for the initial adherence of *E. coli* K-12 to abiotic surfaces [29], and is an important virulence factor in uropathogenic *E. coli* strains [30, 31]. However, T1P of prototype typical EPEC strains E2348/69 and B171 had no effect on LA pattern [32] but were important for development of the AA pattern on EAEC strain 042 [33]. The *traJ* gene regulates the expression of F pilus involved in bacterial conjugation mediated by F plasmids [34]. It has been shown that putative F pilus may work as central adhesion factor during the biofilm formation by typical EAEC strains. In addition, F pili expressed by EAEC strains boosted mixed biofilm formation when in the presence of aggregative *Citrobacter freundii* [35].

The outer membrane invasin and adhesin Tia previously described in enterotoxigenic *E. coli* mediates bacterial attachment to a variety of cultured human epithelial cells, autoaggregation, and biofilm formation [36]. The Hek outer membrane protein of *E. coli* is an auto-aggregating adhesin and invasin reported from uropathogenic *E. coli* and neonatal meningitic *E. coli* [37, 38]. Interestingly, agglutinin genes are common in aggregative *E. coli* (EAEC). EAEC strains harbor the agglutinin genes *hra1* (*hek*), *hra2* and *tia* which confer autoaggregation, biofilm formation, and AA patterns [39, 40]. In this study, we were able to demonstrate that these agglutinin genes were encoded on the pIS102 plasmid. By using the Tn5 mutants as donors, we were able to obtain kanamycin-resistant *E. coli* MA3456 transconjugants. Sequence analysis of the plasmid genes revealed a region exhibit to 80 and 73% amino acid similarities to the agglutinins Tia and Hek, respectively. Future efforts will be directed to construct a *tia/kek* homolog deletion mutant of A102 strain.

## Conclusions

In this study, we have identified three large conjugative plasmids, pIS46, pIS66 and pIS102, coding for antimicrobial resistance and localized-like adherence (LAL) to HeLa cells. In addition, we identified a *tia*/*hek* homolog encoded on the pIS102 plasmid, which seems to be involved in adhesion of A102 strain. Characterization of this *tia*/*hek* homolog may bring new insights into aEPEC colonization.

## Methods

### Bacterial strains

Eighteen aEPEC strains showing LAL and belonging to different serotypes were studied [23]. All strains were isolated from children with diarrhea. The *E. coli* K-12 strains C600, J53 and DHα5, and *E. coli* MA3456 were used as the recipient for EPEC plasmids during conjugation or transformation experiments. *E. coli* DH5αλ*pir* was used for cloning studies. The strains were cultured in Luria-Bertani (LB) broth or on LB agar at 37 °C with appropriate antibiotics when necessary.

### Antimicrobial susceptibility testing

Antimicrobial susceptibility tests were performed by the disk diffusion method [41] using disks containing ampicillin (10 μg), chloramphenicol (30 μg), kanamycin (30 μg), nalidixic acid (30 μg), sulfonamide (300 μg), streptomycin (10 μg), and tetracycline (30 μg). The inhibition zone diameters were interpreted according to Clinical and Laboratory Standards Institute requirements [41], and the *E. coli* NCTC10418 was used as the control.

### HeLa cell adherence assay

The HeLa cell adhesion assay was performed as previously described [12] with modifications. Briefly, monolayers of 10^5^ HeLa cells were grown in Dulbecco modified Eagle medium containing 10% fetal bovine serum, by use of 24-well tissue culture plates. Bacteria strains were grown statically in 2 ml of LB for 16–18 h at 37 °C. Cell monolayers were infected with 20 μl of bacterial cultures added to 1 ml of DMEM and were incubated at 37 °C for 3 h. After incubation, the cells were washed with sterile PBS, fixed with methanol, stained with Giemsa stain, and examined under a light microscope.

### Plasmid profiling

Plasmid DNA was extracted from overnight bacterial cultures by the alcaline extraction method of Birnboim and Doly [42], and analyzed in 0.8% agarose gels stained with ethidium bromide (5 μg/ml). Plasmid molecular sizes were calculated based on relative migration of plasmids with known sizes contained in strain 39-R861 [24].

### DNA hybridization

Colony and Southern blot hybridization [25] were performed at 65 °C. Gene probes, *traA* [27] were generated by PCR, gel purified, and labeled with [α-^32^P] dCTP using a Rediprime (Amersham Pharmacia Biotech Inc., EUA) according to the manufacturer-s instructions.

### Conjugation experiments

The donor and recipient strains were grown on LB broth to an optical density at 600 nm (OD_600_) of 0.5 mixed equally, and then inoculated on filter papers for 4 h. The filter paper mixtures were then suspended in LB medium, and dilutions were plated on LB agar containing nalidixic acid (50 μg/ml) with either ampicillin (100 μg), kanamycin (50 μg), chloramphenicol (50 μg/ml) or tetracycline (25 μg/ml). Conjugation frequencies were calculated as the ratio of number of transconjugant colonies by the number of donor colonies. Each conjugation experiment was repeated at least twice. The transconjugants were tested for HeLa cell adherence assay as described previously [12].

### Transposon mutagenesis and genetic analysis

Transposon mutants were generated with the kanamycin resistance (Km^r^)-encoding transposome EZ::TN < R6Kγ*ori*/KAN-2> Tnp transposome (Epicentre Biotechnologies) by electroporation according to the manufacturer’s procedures. Briefly, electrocompetent bacterial cells were transformed with 1 μl of the Tnp transposome. Transposon-inserted bacterial colonies that grew on LB agar plates containing nalidixic acid (100 μg/ml), ampicillin (100 μg/ml) and kanamycin (50 μg/ml) were screened for their adhesion phenotype to HeLa cells as described below. Genomic DNA was isolated from mutants by using the PureLink Genomic DNA Mini kit (Invitrogen). Genomic DNA of the mutants, was digested with *Eco*RI, self-ligated by the addition of T4 DNA ligase, and then used for transformation of *E. coli* DH5αλ*pir*. Rescued DNA plasmids were purified by using the Mini plasmid kit (Qiagen) and sequenced by using transposon-specific primers R6KAN-2 RP-1 and KAN-2 FP-1 (Epicentre). DNA sequencing was performed at the Centro de Estudos do Genoma Humano-USP, São Paulo. Nucleotide sequence data were analyzed using SeqMan and MegAlign software and the BLAST tool (http://www.ncbi.nlm.nih.gov/BLAST).

## Supplementary information

**Additional file 1 **: **Figure S1** BLAST results of the 1175 bp sequence obtained from transconjugant II-A-7. Description of data: Descriptions and alignments of the 1175 pb sequence.

## Data Availability

The data is available upon request. Please contact the corresponding author Isabel C A Scaletsky, E-mail: scaletskyunifesp@gmail.com

## Abbreviations

EPEC: Enteropathogenic *Escherichia coli*
tEPEC: typical EPEC
aEPEC: atypical EPEC
LA: Localized adherence
LAL: Localized adherence-like
DA: Diffuse adherence
AA: Aggregative adherence
EAF: *E. coli* adherence factor plasmid
BFP: Bundle-forming pilus
TIP: Type 1 pili
