# Supplementary material for: Large plasmids encoding antibiotic resistance and localized-like adherence in atypical enteropathogenic Escherichia coli strains
Source: BMC Microbiol. 2020 May 29;20:138. doi: 10.1186/s12866-020-01809-4 (PMC7257209; doi:10.1186/s12866-020-01809-4)
Supplement: Supplementary file 1 — Additional file 1 : Figure S1 BLAST results of the 1175 bp sequence obtained from transconjugant II-A-7. Description of data: Descriptions and alignments of the 1175 pb sequence. [file 12866_2020_1809_MOESM1_ESM.pdf]

BLAST Results

Nucleotide Sequence (1175 letters)

**RID**	MSG2A4MC01R (Expires on 05-31 21:29 pm)
**Query ID**	lcl|Query_361808
**Description**	None
**Molecule type**	nucleic acid
**Query Length**	1175
**Database Name**	nr
**Description**	All non-redundant GenBank CDS translations+PDB+SwissProt+PIR+PRF excluding environmental samples from WGS projects
**Program**	BLASTX 2.3.1+

Graphic Summary

No putative conserved domains have been detected

Distribution of 100 Blast Hits on the Query Sequence

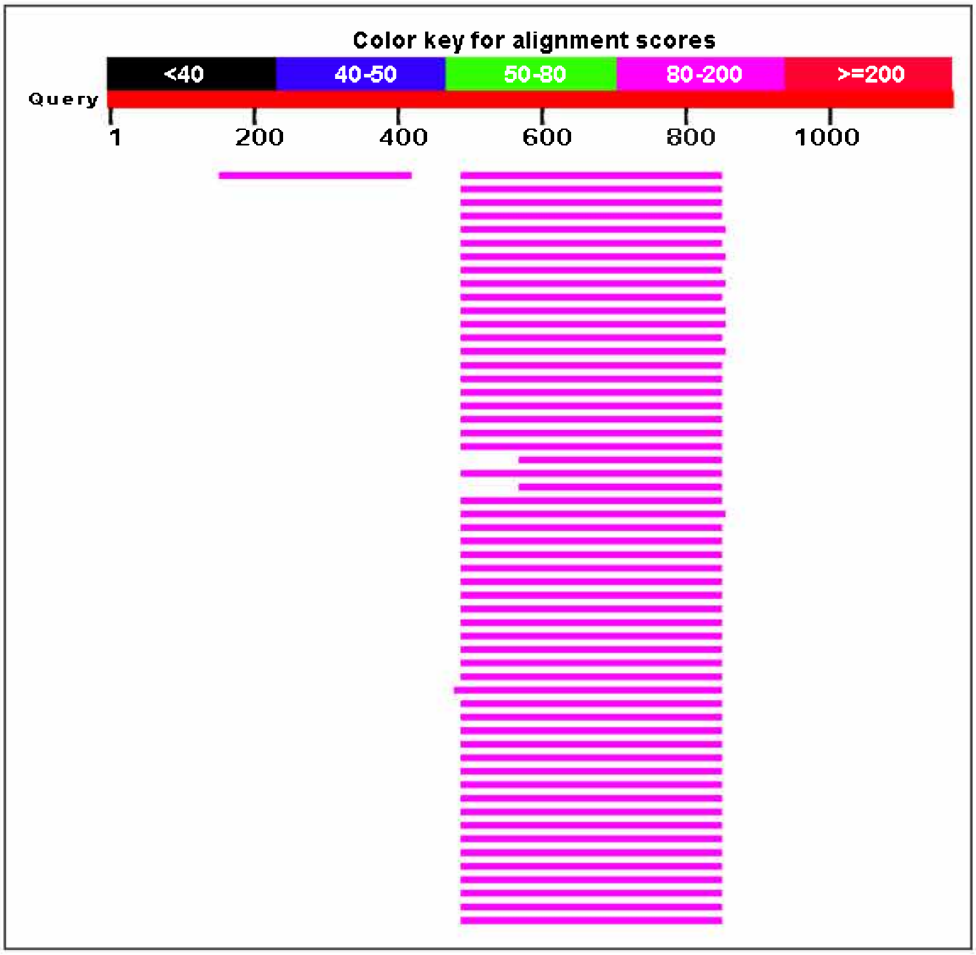

Descriptions

Sequences producing significant alignments:

Description	Max score	Total score	Query cover	E value	Ident	Accession
hypothetical protein [Escherichia coli]	193	193	30%	5e-57	80%	WP_044069946.1
hypothetical protein [Escherichia coli]	193	193	30%	8e-57	80%	WP_049144422.1
tia invasion determinant domain protein [Escherichia coli]	193	193	30%	9e-57	80%	WP_042194856.1
tia invasion determinant domain protein [Escherichia coli]	193	193	30%	1e-56	80%	WP_050436733.1
draP [Escherichia coli LAU-EC10]	174	174	22%	8e-51	96%	ETE17982.1
tia invasion determinant domain protein [Escherichia coli DEC13E]	175	175	31%	6e-50	72%	EHX71337.1
hypothetical protein G770_04687 [Escherichia coli HVH 109 (4-6977162)]	173	173	30%	8e-50	72%	EQQ62779.1
adhesin [Escherichia coli]	177	177	31%	9e-50	73%	WP_000571846.1
hypothetical protein [Escherichia coli]	174	174	30%	9e-50	72%	WP_058033427.1
tia invasion determinant [Escherichia coli MS 69-1]	176	176	31%	1e-49	73%	EFJ81905.1
outer membrane beta-barrel domain protein [Escherichia coli]	176	176	30%	1e-49	73%	WP_001380747.1
adhesin [Escherichia coli LAU-EC10]	176	176	31%	1e-49	73%	ETE23623.1
ompA-like transmembrane domain protein [Escherichia coli DEC14C]	176	176	31%	1e-49	73%	EHX85258.1
adhesin [Escherichia coli]	176	176	30%	1e-49	73%	WP_061330160.1
tia invasion determinant [Escherichia coli ETEC H10407]	176	176	31%	1e-49	73%	AAB06592.1
adhesin/virulence factor Hek [Escherichia coli]	176	176	30%	1e-49	73%	WP_001601288.1
adhesin/virulence factor Hek [Escherichia coli]	176	176	30%	2e-49	72%	WP_021536698.1
adhesin [Escherichia coli]	174	174	30%	2e-49	72%	WP_044074289.1
adhesin [Escherichia coli]	175	175	30%	2e-49	72%	WP_059327261.1
adhesin [Escherichia coli]	174	174	30%	2e-49	72%	WP_042098513.1
adhesin [Escherichia coli]	175	175	30%	3e-49	72%	WP_059333072.1
tia invasion determinant domain protein [Escherichia coli]	173	173	30%	3e-49	72%	WP_001438278.1
hypothetical protein G768_04025 [Escherichia coli HVH 107 (4-5860571)]	171	171	24%	3e-49	88%	EQQ66869.1
adhesin [Escherichia coli]	174	174	30%	3e-49	72%	WP_059341043.1
hypothetical protein PU18_24840 [Escherichia coli]	171	171	24%	3e-49	88%	KHI75040.1
adhesin [Escherichia coli]	175	175	30%	3e-49	72%	WP_047626560.1
tia invasion determinant [Escherichia coli 99.0741]	176	176	31%	4e-49	73%	EIH45503.1
outer membrane beta-barrel domain protein [Escherichia coli]	174	174	30%	5e-49	72%	WP_001407647.1
adhesin [Escherichia coli]	175	175	30%	5e-49	72%	WP_061361977.1
outer membrane beta-barrel domain protein [Escherichia coli]	174	174	30%	5e-49	72%	WP_001412695.1
adhesin/virulence factor Hek [Escherichia coli UMEA 3185-1]	176	176	30%	6e-49	72%	EQX63545.1
adhesin/virulence factor Hek						

[Escherichia coli]	174	174	30%	6e-49	72%	WP_001774679.1
outer membrane protein PagN [Escherichia coli]	174	174	30%	6e-49	72%	WP_001738403.1
MULTISPECIES: adhesin [Escherichia]	174	174	30%	6e-49	72%	WP_001350744.1
MULTISPECIES: adhesin/virulence factor Hek [Escherichia]	174	174	30%	6e-49	72%	WP_001572253.1
adhesin [Escherichia coli]	174	174	30%	6e-49	72%	WP_032295294.1
adhesin [Escherichia coli]	174	174	30%	6e-49	72%	WP_031326373.1
adhesin [Escherichia coli]	174	174	30%	7e-49	72%	WP_042098515.1
outer membrane protein PagN [Escherichia coli]	174	174	30%	7e-49	72%	WP_001486234.1
adhesin [Escherichia coli]	174	174	31%	7e-49	73%	WP_001406302.1
adhesin [Escherichia coli]	174	174	30%	7e-49	72%	WP_032326905.1
outer membrane protein PagN [Escherichia coli]	174	174	30%	7e-49	72%	WP_001485097.1
adhesin/virulence factor Hek [Escherichia coli]	174	174	30%	8e-49	72%	WP_021538217.1
adhesin [Escherichia coli]	174	174	30%	8e-49	72%	WP_032175896.1
adhesin [Escherichia coli]	174	174	30%	8e-49	72%	WP_059319669.1
adhesin [Escherichia coli]	174	174	30%	9e-49	72%	WP_001536427.1
adhesin [Escherichia coli]	174	174	30%	9e-49	72%	WP_061892651.1
adhesin [Escherichia coli]	174	174	30%	9e-49	72%	WP_000719909.1
adhesin [Escherichia coli]	174	174	30%	9e-49	72%	WP_001318118.1
tia protein [Escherichia coli TA007]	174	174	30%	1e-48	72%	EGB64410.1
adhesin [Escherichia coli]	174	174	30%	1e-48	72%	WP_033805161.1
adhesin [Escherichia coli]	174	174	30%	1e-48	72%	WP_062871888.1
hypothetical protein [Escherichia coli]	174	174	30%	1e-48	72%	WP_000719910.1
adhesin [Escherichia coli]	174	174	30%	1e-48	72%	WP_061358567.1
adhesin [Escherichia coli]	173	173	30%	2e-48	72%	WP_032208082.1
adhesin/virulence factor Hek [Escherichia coli]	173	173	30%	3e-48	72%	WP_001588944.1
adhesin [Escherichia coli]	173	173	30%	3e-48	72%	WP_040074753.1
adhesin [Escherichia coli]	171	171	30%	3e-48	72%	WP_042058673.1
adhesin [Escherichia coli]	172	172	30%	3e-48	72%	WP_029488461.1
adhesin [Escherichia coli]	172	172	30%	4e-48	72%	WP_032198288.1
adhesin [Escherichia coli]	172	172	24%	5e-48	88%	WP_057697893.1
adhesin [Escherichia coli]	172	172	30%	5e-48	72%	WP_047664222.1
hypothetical protein [Escherichia coli]	172	172	30%	6e-48	72%	WP_000719911.1
adhesin [Escherichia coli]	172	172	30%	6e-48	72%	WP_063115501.1
tia invasion determinant [Escherichia coli 1.2264]	173	173	31%	8e-48	72%	EIH22380.1
adhesin/virulence factor Hek [Escherichia coli]	171	171	30%	8e-48	72%	WP_001608785.1
adhesin [Escherichia coli]	171	171	30%	1e-47	72%	WP_028131540.1
adhesin [Escherichia coli]	171	171	30%	1e-47	72%	WP_062893151.1
adhesin [Escherichia coli]	171	171	30%	2e-47	71%	WP_063103299.1
adhesin [Escherichia coli]	171	171	30%	2e-47	72%	WP_000719907.1
adhesin [Escherichia coli]	170	170	30%	3e-47	72%	WP_032261250.1
hypothetical protein PU18_25750 [Escherichia coli]	166	166	24%	3e-47	85%	KHI72541.1
adhesin [Escherichia coli]	169	169	30%	7e-47	70%	WP_001385633.1
tia invasion determinant [Escherichia coli]	169	169	30%	8e-47	71%	AFX83958.1
ompA-like transmembrane domain protein [Escherichia coli E128010]	169	169	31%	1e-46	69%	EFZ46709.1

adhesin [Escherichia coli]	169	169	30%	1e-46	70%	WP_042342646.1
adhesin [Escherichia coli]	168	168	30%	1e-46	71%	WP_032219902.1
adhesin [Escherichia coli]	168	168	30%	2e-46	71%	WP_033884667.1
adhesin/virulence factor Hek [Escherichia coli]	168	168	31%	2e-46	69%	WP_021558531.1
adhesin [Escherichia coli]	168	168	30%	2e-46	71%	WP_032199216.1
outer membrane protein PagN [Escherichia coli]	167	167	30%	4e-46	69%	WP_001757780.1
adhesin [Escherichia coli]	167	167	30%	4e-46	69%	WP_027662028.1
adhesin [Escherichia coli]	167	167	30%	4e-46	69%	WP_024184564.1
adhesin [Escherichia coli]	167	167	30%	6e-46	71%	WP_032293016.1
adhesin [Escherichia coli]	166	166	30%	1e-45	70%	WP_032221534.1
tia invasion determinant [Escherichia coli 9.0111]	167	167	31%	2e-45	70%	EII24851.1
adhesin [Escherichia coli]	166	166	30%	2e-45	70%	WP_042960748.1
adhesin [Escherichia coli]	165	165	30%	3e-45	68%	WP_052895034.1
adhesin [Escherichia coli]	165	165	30%	3e-45	70%	WP_032223544.1
hypothetical protein [Escherichia coli]	164	164	30%	5e-45	69%	WP_000719908.1
adhesin [Escherichia coli]	161	161	31%	6e-44	68%	WP_032186291.1
adhesin [Escherichia coli]	159	159	30%	5e-43	67%	WP_032229323.1
adhesin [Escherichia coli]	156	156	30%	8e-42	66%	WP_032290946.1
tia invasion determinant [Escherichia coli MS 84-1]	149	149	24%	4e-39	78%	EFJ85770.1
adhesin [Escherichia coli]	149	149	24%	5e-39	79%	WP_061350558.1
adhesin [Escherichia coli]	148	148	24%	6e-39	79%	WP_041124155.1
Putative peptide transport periplasmic protein [Escherichia coli NA114]	148	148	24%	1e-38	77%	AEG39517.1
tia invasion determinant [Escherichia coli 113290]	148	148	24%	1e-38	77%	ESA68633.1
tia invasion determinant [Escherichia coli DEC6B]	147	147	24%	1e-38	77%	EHV50316.1
tia invasion determinant [Escherichia coli 908573]	147	147	24%	1e-38	77%	ESD79942.1

Alignments

hypothetical protein, partial [Escherichia coli]

Sequence ID: **ref|WP_044069946.1|** Length: 183 Number of Matches: 1

Range 1: 1 to 123

Score	Expect	Method	Identities	Positives	Gaps	Frame
193 bits(491)	5e-57()	Compositional matrix adjust.	99/123(80%)	103/123(83%)	2/123(1%)	-1

Features:

```
Query  854   MKKIITVSAMAMAGMFSTQTLADEGKTGFYVTGKAGASVVTQIDQRFRQDFGDVVYKYKG  675
             MKKIITVSAMAMAGMFSTQTLADEGKTGFYVTGKAGASVVTQIDQRFRQDFGDVVYKYKG
Sbjct  1     MKKIITVSAMAMAGMFSTQTLADEGKTGFYVTGKAGASVVTQIDQRFRQDFGDVVYKYKG  60

Query  674   SDKNDTVFGAGLAVGYDFYQHYNVPVRTEVEFTGGSDLYSAEEAEGYLQP--GGGTQGIQ  501
             SDKNDTVFGAGLAVGYDFYQHYNVPVRTEVEF G     S    + +  P   GG +  Q
Sbjct  61    SDKNDTVFGAGLAVGYDFYQHYNVPVRTEVEFYGRGAADSRYTLDTWRSPMGDGGREDTQ  120

Query  500   ERI  492
              R+
Sbjct  121   NRL  123
```

hypothetical protein, partial [Escherichia coli]

Sequence ID: **ref|WP_049144422.1|** Length: 191 Number of Matches: 1

Range 1: 1 to 123

Score	Expect	Method	Identities	Positives	Gaps	Frame
193 bits(490)	8e-57()	Compositional matrix adjust.	99/123(80%)	103/123(83%)	2/123(1%)	-1

Features:

```
Query  854  MKKIITVSAMAMAGMFSTQTLADEGKTGFYVTGKAGASVVTQIDQRFRQDFGDVVYKYKG  675
            MKKIITVSAMAMAGMFSTQTLADEGKTGFYVTGKAGASVVTQIDQRFRQDFGDVVYKYKG
Sbjct  1    MKKIITVSAMAMAGMFSTQTLADEGKTGFYVTGKAGASVVTQIDQRFRQDFGDVVYKYKG  60

Query  674  SDKNDTVFGAGLAVGYDFYQHYNVPVRTEVEFTGGSDLYSAEEAEGYLQP--GGGTQGIQ  501
            SDKNDTVFGAGLAVGYDFYQHYNVPVRTEVEF G     S    + +  P   GG +  Q
Sbjct  61   SDKNDTVFGAGLAVGYDFYQHYNVPVRTEVEFYGRGAADSRYTLDTWRSPMGDGGREDTQ  120

Query  500  ERI  492
             R+
Sbjct  121  NRL  123
```

tia invasion determinant domain protein [Escherichia coli]
Sequence ID: **ref|WP_042194856.1|** Length: 199 Number of Matches: 1
Range 1: 1 to 123

Score	Expect	Method	Identities	Positives	Gaps	Frame
193 bits(490)	9e-57()	Compositional matrix adjust.	99/123(80%)	103/123(83%)	2/123(1%)	-1

Features:

```
Query  854  MKKIITVSAMAMAGMFSTQTLADEGKTGFYVTGKAGASVVTQIDQRFRQDFGDVVYKYKG  675
            MKKIITVSAMAMAGMFSTQTLADEGKTGFYVTGKAGASVVTQIDQRFRQDFGDVVYKYKG
Sbjct  1    MKKIITVSAMAMAGMFSTQTLADEGKTGFYVTGKAGASVVTQIDQRFRQDFGDVVYKYKG  60

Query  674  SDKNDTVFGAGLAVGYDFYQHYNVPVRTEVEFTGGSDLYSAEEAEGYLQP--GGGTQGIQ  501
            SDKNDTVFGAGLAVGYDFYQHYNVPVRTEVEF G     S    + +  P   GG +  Q
Sbjct  61   SDKNDTVFGAGLAVGYDFYQHYNVPVRTEVEFYGRGAADSRYTLDTWRSPMGDGGREDTQ  120

Query  500  ERI  492
             R+
Sbjct  121  NRL  123
```

tia invasion determinant domain protein [Escherichia coli]
Sequence ID: **ref|WP_050436733.1|** Length: 199 Number of Matches: 1

See 1 more title(s)
Range 1: 1 to 123

Score	Expect	Method	Identities	Positives	Gaps	Frame
193 bits(490)	1e-56()	Compositional matrix adjust.	99/123(80%)	103/123(83%)	2/123(1%)	-1

Features:

```
Query  854  MKKIITVSAMAMAGMFSTQTLADEGKTGFYVTGKAGASVVTQIDQRFRQDFGDVVYKYKG  675
            MKKIITVSAMAMAGMFSTQTLADEGKTGFYVTGKAGASVVTQIDQRFRQDFGDVVYKYKG
Sbjct  1    MKKIITVSAMAMAGMFSTQTLADEGKTGFYVTGKAGASVVTQIDQRFRQDFGDVVYKYKG  60

Query  674  SDKNDTVFGAGLAVGYDFYQHYNVPVRTEVEFTGGSDLYSAEEAEGYLQP--GGGTQGIQ  501
            SDKNDTVFGAGLAVGYDFYQHYNVPVRTEVEF G     S    + +  P   GG +  Q
Sbjct  61   SDKNDTVFGAGLAVGYDFYQHYNVPVRTEVEFYGRGAADSRYTLDTWRSPMGDGGREDTQ  120

Query  500  ERI  492
             R+
Sbjct  121  NRL  123
```

draP [Escherichia coli LAU-EC10]
Sequence ID: **gb|ETE17982.1|** Length: 89 Number of Matches: 1
Range 1: 1 to 89

Score	Expect	Method	Identities	Positives	Gaps	Frame
174 bits(440)	8e-51()	Compositional matrix adjust.	85/89(96%)	86/89(96%)	0/89(0%)	-3

Features:

```
Query  423  VLAVVYYININRPSGFNLNSQNHLNQKQGGGFIMGNLPVGSRGLYKGKKRSAGMNESRHPG  244
            +LAVVYYININRPSGFNLNSQNHLNQKQGGGFIMGNLPVGSRGLYKGKKRSAGMNE RHPG
Sbjct  1    MLAVVYYININRPSGFNLNSQNHLNQKQGGGFIMGNLPVGSRGLYKGKKRSAGMNELRHPG  60

Query  243  STQMTCTGQAHRGGEHRLNTGPPDWQTVE  157
            STQMT TGQAHRGGEH LNTGPPDWQTVE
Sbjct  61   STQMTRTGQAHRGGEHWLNTGPPDWQTVE  89
```

tia invasion determinant domain protein [Escherichia coli DEC13E]
Sequence ID: **gb|EHX71337.1|** Length: 188 Number of Matches: 1
Range 1: 3 to 126

Score	Expect	Method	Identities	Positives	Gaps	Frame
175 bits(444)	6e-50()	Compositional matrix adjust.	89/124(72%)	97/124(78%)	2/124(1%)	-1

Features:

```
Query  857   EMKKIITVSAMAMAGMFSTQTLADEGKTGFYVTGKAGASVVTQIDQRFRQDFGDVVYKYK  678
             EMKK+I VSA+AMAG+FS Q LAD GKTGFYVTGKAGAS+VTQ DQRFRQDFGD VYKYK
Sbjct  3     EMKKVIAVSALAMAGVFSAQALADRGKTGFYVTGKAGASIVTQTDQRFRQDFGDDVYKYK  62

Query  677   GSDKNDTVFGAGLAVGYDFYQHYNVPVRTEVEFTGGSDLYSAEEAEGYLQP--GGGTQGI  504
             G DKNDTVFGAGLAVGYDFYQHYNVPVRTEVEF G     S    + +  P   GG +
Sbjct  63    GGDKNDTVFGAGLAVGYDFYQHYNVPVRTEVEFYGRGAADSRYTLDTWHSPMGDGGREDT  122

Query  503   QERI  492
             Q R+
Sbjct  123   QNRL  126
```

hypothetical protein G770_04687 [Escherichia coli HVH 109 (4-6977162)]

Sequence ID: **gb|EQQ62779.1|** Length: 138 Number of Matches: 1

Range 1: 1 to 123

Score	Expect	Method	Identities	Positives	Gaps	Frame
173 bits(438)	8e-50()	Compositional matrix adjust.	89/123(72%)	96/123(78%)	2/123(1%)	-1

Features:

```
Query  854   MKKIITVSAMAMAGMFSTQTLADEGKTGFYVTGKAGASVVTQIDQRFRQDFGDVVYKYKG  675
             MKK+I VSA+AMAG+FS Q LAD GKTGFYVTGKAGASVVTQ DQRFRQDFGD VYKYKG
Sbjct  1     MKKVIVVSALAMAGVFSAQALADRGKTGFYVTGKAGASVVTQTDQRFRQDFGDDVYKYKG  60

Query  674   SDKNDTVFGAGLAVGYDFYQHYNVPVRTEVEFTGGSDLYSAEEAEGYLQP--GGGTQGIQ  501
              DKNDTVFGAGLAVGYDFYQHYNVPVRTEVEF G     S    + +  P   GG +  Q
Sbjct  61    GDKNDTVFGAGLAVGYDFYQHYNVPVRTEVEFYGRGAADSHYTLDTWHSPMGDGGREDTQ  120

Query  500   ERI  492
              R+
Sbjct  121   NRL  123
```

adhesin [Escherichia coli]

Sequence ID: **ref|WP_000571846.1|** Length: 251 Number of Matches: 1

See 13 more title(s)

Range 1: 3 to 126

Score	Expect	Method	Identities	Positives	Gaps	Frame
177 bits(448)	9e-50()	Compositional matrix adjust.	90/124(73%)	97/124(78%)	2/124(1%)	-1

Features:

```
Query  857   EMKKIITVSAMAMAGMFSTQTLADEGKTGFYVTGKAGASVVTQIDQRFRQDFGDVVYKYK  678
             EMKK+I VSA+AMAG+FS Q LAD GKTGFYVTGKAGASVVTQ DQRFRQDFGD VYKYK
Sbjct  3     EMKKVIVVSALAMAGVFSAQALADRGKTGFYVTGKAGASVVTQTDQRFRQDFGDDVYKYK  62

Query  677   GSDKNDTVFGAGLAVGYDFYQHYNVPVRTEVEFTGGSDLYSAEEAEGYLQP--GGGTQGI  504
             G DKNDTVFGAGLAVGYDFYQHYNVPVRTEVEF G     S    + +  P   GG +
Sbjct  63    GGDKNDTVFGAGLAVGYDFYQHYNVPVRTEVEFYGRGAADSHYTLDTWHSPMGDGGREDT  122

Query  503   QERI  492
             Q R+
Sbjct  123   QNRL  126
```

hypothetical protein, partial [Escherichia coli]

Sequence ID: **ref|WP_058033427.1|** Length: 181 Number of Matches: 1

Range 1: 1 to 123

Score	Expect	Method	Identities	Positives	Gaps	Frame
174 bits(442)	9e-50()	Compositional matrix adjust.	89/123(72%)	96/123(78%)	2/123(1%)	-1

Features:

```
Query  854   MKKIITVSAMAMAGMFSTQTLADEGKTGFYVTGKAGASVVTQIDQRFRQDFGDVVYKYKG  675
             MKK+I VSA+AMAG+FS Q LAD GKTGFYVTGKAGASVVTQ DQRFRQDFGD VYKYKG
Sbjct  1     MKKVIVVSALAMAGVFSAQALADRGKTGFYVTGKAGASVVTQTDQRFRQDFGDDVYKYKG  60

Query  674   SDKNDTVFGAGLAVGYDFYQHYNVPVRTEVEFTGGSDLYSAEEAEGYLQP--GGGTQGIQ  501
              DKNDTVFGAGLAVGYDFYQHYNVPVRTEVEF G     S    + +  P   GG +  Q
Sbjct  61    GDKNDTVFGAGLAVGYDFYQHYNVPVRTEVEFYGRGAADSHYTLDTWHSPMGDGGREDTQ  120

Query  500   ERI  492
              R+
Sbjct  121   NRL  123
```

tia invasion determinant [Escherichia coli MS 69-1]

Sequence ID: **gb|EFJ81905.1|** Length: 251 Number of Matches: 1

Range 1: 3 to 126

Score	Expect	Method	Identities	Positives	Gaps	Frame

176 bits(447)	1e-49()	Compositional matrix adjust.	90/124(73%)	97/124(78%)	2/124(1%)	-1

Features:

```
Query  857   EMKKIITVSAMAMAGMFSTQTLADEGKTGFYVTGKAGASVVTQIDQRFRQDFGDVVYKYK  678
             EMKK+I VSA+AMAG+FS Q LAD GKTGFYVTGKAGASVVTQ DQRFRQDFGD VYKYK
Sbjct  3     EMKKVIVVSALAMAGVFSAQALADRGKTGFYVTGKAGASVVTQTDQRFRQDFGDDVYKYK  62

Query  677   GSDKNDTVFGAGLAVGYDFYQHYNVPVRTEVEFTGGSDLYSAEEAEGYLQP--GGGTQGI  504
             G DKNDTVFGAGLAVGYDFYQHYNVPVRTEVEF G     S    + +  P   GG +
Sbjct  63    GGDKNDTVFGAGLAVGYDFYQHYNVPVRTEVEFYGRGAADSHYTLDTWHSPMGDGGREDT  122

Query  503   QERI  492
             Q R+
Sbjct  123   QNRL  126
```

outer membrane beta-barrel domain protein, partial [Escherichia coli]

Sequence ID: **ref|WP_001380747.1|** Length: 244 Number of Matches: 1

See 1 more title(s)

Range 1: 1 to 123

Score	Expect	Method	Identities	Positives	Gaps	Frame
176 bits(447)	1e-49()	Compositional matrix adjust.	90/123(73%)	96/123(78%)	2/123(1%)	-1

Features:

```
Query  854   MKKIITVSAMAMAGMFSTQTLADEGKTGFYVTGKAGASVVTQIDQRFRQDFGDVVYKYKG  675
             MKK+I VSA+AMAGMFS QTLADE KTGFYVTGKAGASVV Q DQRFRQDFGD VYKYKG
Sbjct  1     MKKVIAVSALAMAGMFSAQTLADESKTGFYVTGKAGASVVMQTDQRFRQDFGDDVYKYKG  60

Query  674   SDKNDTVFGAGLAVGYDFYQHYNVPVRTEVEFTGGSDLYSAEEAEGYLQP--GGGTQGIQ  501
              DKNDTVFGAGLAVGYDFYQHYNVPVRTEVEF G     S    + +  P   GG +  Q
Sbjct  61    GDKNDTVFGAGLAVGYDFYQHYNVPVRTEVEFYGRGAADSRYTLDTWRSPMGDGGREDTQ  120

Query  500   ERI  492
              R+
Sbjct  121   NRL  123
```

adhesin [Escherichia coli LAU-EC10]

Sequence ID: **gb|ETE23623.1|** Length: 251 Number of Matches: 1

Range 1: 3 to 126

Score	Expect	Method	Identities	Positives	Gaps	Frame
176 bits(447)	1e-49()	Compositional matrix adjust.	90/124(73%)	97/124(78%)	2/124(1%)	-1

Features:

```
Query  857   EMKKIITVSAMAMAGMFSTQTLADEGKTGFYVTGKAGASVVTQIDQRFRQDFGDVVYKYK  678
             EMKK+I VSA+AMAG+FS Q LAD GKTGFYVTGKAGASVVTQ DQRFRQDFGD VYKYK
Sbjct  3     EMKKVIVVSALAMAGVFSAQALADRGKTGFYVTGKAGASVVTQTDQRFRQDFGDDVYKYK  62

Query  677   GSDKNDTVFGAGLAVGYDFYQHYNVPVRTEVEFTGGSDLYSAEEAEGYLQP--GGGTQGI  504
             G DKNDTVFGAGLAVGYDFYQHYNVPVRTEVEF G     S    + +  P   GG +
Sbjct  63    GGDKNDTVFGAGLAVGYDFYQHYNVPVRTEVEFYGRGAADSHYTLDTWHSPMGDGGREDT  122

Query  503   QERI  492
             Q R+
Sbjct  123   QNRL  126
```

ompA-like transmembrane domain protein [Escherichia coli DEC14C]

Sequence ID: **gb|EHX85258.1|** Length: 251 Number of Matches: 1

Range 1: 3 to 126

Score	Expect	Method	Identities	Positives	Gaps	Frame
176 bits(447)	1e-49()	Compositional matrix adjust.	90/124(73%)	96/124(77%)	2/124(1%)	-1

Features:

```
Query  857   EMKKIITVSAMAMAGMFSTQTLADEGKTGFYVTGKAGASVVTQIDQRFRQDFGDVVYKYK  678
             EMKK+I VSA+AMAGMFS Q LADE KTGFYVTGKAGASVV Q DQRFRQDFGD VYKYK
Sbjct  3     EMKKVIAVSALAMAGMFSAQALADESKTGFYVTGKAGASVVMQTDQRFRQDFGDDVYKYK  62

Query  677   GSDKNDTVFGAGLAVGYDFYQHYNVPVRTEVEFTGGSDLYSAEEAEGYLQP--GGGTQGI  504
             G DKNDTVFGAGLAVGYDFYQHYNVPVRTEVEF G     S    + +  P   GG +
Sbjct  63    GGDKNDTVFGAGLAVGYDFYQHYNVPVRTEVEFYGRGAADSRYTLDTWRSPMGDGGREDT  122

Query  503   QERI  492
             Q R+
Sbjct  123   QNRL  126
```

adhesin [Escherichia coli]

Sequence ID: **ref|WP_061330160.1|** Length: 248 Number of Matches: 1

See 4 more title(s)
Range 1: 1 to 123

Score	Expect	Method	Identities	Positives	Gaps	Frame
176 bits(446)	1e-49()	Compositional matrix adjust.	90/123(73%)	96/123(78%)	2/123(1%)	-1

Features:

```
Query  854   MKKIITVSAMAMAGMFSTQTLADEGKTGFYVTGKAGASVVTQIDQRFRQDFGDVVYKYKG  675
             MKK+I VSA+AMAGMFS Q LADE KTGFYVTGKAGASVVTQ DQRFRQDFGD VYKYKG
Sbjct  1     MKKVIAVSALAMAGMFSAQVLADESKTGFYVTGKAGASVVTQTDQRFRQDFGDDVYKYKG  60

Query  674   SDKNDTVFGAGLAVGYDFYQHYNVPVRTEVEFTGGSDLYSAEEAEGYLQP--GGGTQGIQ  501
              DKNDTVFGAGLAVGYDFYQHYNVPVRTEVEF G     S    + +  P   GG +  Q
Sbjct  61    GDKNDTVFGAGLAVGYDFYQHYNVPVRTEVEFYGRGAADSRYTLDTWHSPMGDGGREDTQ  120

Query  500   ERI  492
              R+
Sbjct  121   NRL  123
```

tia invasion determinant [Escherichia coli ETEC H10407]
Sequence ID: **gb|AAB06592.1|** Length: 251 Number of Matches: 1
Range 1: 3 to 126

Score	Expect	Method	Identities	Positives	Gaps	Frame
176 bits(447)	1e-49()	Compositional matrix adjust.	90/124(73%)	96/124(77%)	2/124(1%)	-1

Features:

```
Query  857   EMKKIITVSAMAMAGMFSTQTLADEGKTGFYVTGKAGASVVTQIDQRFRQDFGDVVYKYK  678
             EMKK+I VSA+AMAGMFS Q LADE KTGFYVTGKAGASVV Q DQRFRQDFGD VYKYK
Sbjct  3     EMKKVIAVSALAMAGMFSAQALADESKTGFYVTGKAGASVVMQTDQRFRQDFGDDVYKYK  62

Query  677   GSDKNDTVFGAGLAVGYDFYQHYNVPVRTEVEFTGGSDLYSAEEAEGYLQP--GGGTQGI  504
             G DKNDTVFGAGLAVGYDFYQHYNVPVRTEVEF G     S    + +  P   GG +
Sbjct  63    GGDKNDTVFGAGLAVGYDFYQHYNVPVRTEVEFYGRGAADSRYTLDTWRSPMGDGGREDT  122

Query  503   QERI  492
             Q R+
Sbjct  123   QNRL  126
```

adhesin/virulence factor Hek [Escherichia coli]
Sequence ID: **ref|WP_001601288.1|** Length: 248 Number of Matches: 1

See 1 more title(s)
Range 1: 1 to 123

Score	Expect	Method	Identities	Positives	Gaps	Frame
176 bits(446)	1e-49()	Compositional matrix adjust.	90/123(73%)	96/123(78%)	2/123(1%)	-1

Features:

```
Query  854   MKKIITVSAMAMAGMFSTQTLADEGKTGFYVTGKAGASVVTQIDQRFRQDFGDVVYKYKG  675
             MKK+I VSA+AMAGMFS QTLADE KTGFYVTGKAGASVV Q DQRFRQDFGD VYKYKG
Sbjct  1     MKKVIAVSALAMAGMFSAQTLADENKTGFYVTGKAGASVVNQSDQRFRQDFGDDVYKYKG  60

Query  674   SDKNDTVFGAGLAVGYDFYQHYNVPVRTEVEFTGGSDLYSAEEAEGYLQP--GGGTQGIQ  501
              DKNDTVFGAGLAVGYDFYQHYNVPVRTEVEF G     S    + +  P   GG +  Q
Sbjct  61    GDKNDTVFGAGLAVGYDFYQHYNVPVRTEVEFYGRGAADSRYTLDTWHSPMGDGGQEDTQ  120

Query  500   ERI  492
              R+
Sbjct  121   NRL  123
```

adhesin/virulence factor Hek [Escherichia coli]
Sequence ID: **ref|WP_021536698.1|** Length: 248 Number of Matches: 1

See 2 more title(s)
Range 1: 1 to 123

Score	Expect	Method	Identities	Positives	Gaps	Frame
176 bits(445)	2e-49()	Compositional matrix adjust.	89/123(72%)	97/123(78%)	2/123(1%)	-1

Features:

```
Query  854   MKKIITVSAMAMAGMFSTQTLADEGKTGFYVTGKAGASVVTQIDQRFRQDFGDVVYKYKG  675
             MKK+ITVSA+AMAG+FS Q LAD GKTGFYVTGKAGAS+VTQ DQRFRQDFGD VYKYKG
Sbjct  1     MKKVITVSALAMAGVFSAQALADRGKTGFYVTGKAGASIVTQTDQRFRQDFGDDVYKYKG  60

Query  674   SDKNDTVFGAGLAVGYDFYQHYNVPVRTEVEFTGGSDLYSAEEAEGYLQP--GGGTQGIQ  501
              DKNDTVFGAGLAVGYDFYQHYNVPVRTEVEF G     S    + +  P   GG +  Q
Sbjct  61    GDKNDTVFGAGLAVGYDFYQHYNVPVRTEVEFYGRGAADSRYTLDTWHSPMGDGGREDTQ  120

Query  500   ERI  492
              R+
Sbjct  121   NRL  123
```

adhesin, partial [Escherichia coli]
Sequence ID: **ref|WP_044074289.1|** Length: 205 Number of Matches: 1
Range 1: 1 to 123

Score	Expect	Method	Identities	Positives	Gaps	Frame
174 bits(442)	2e-49()	Compositional matrix adjust.	89/123(72%)	96/123(78%)	2/123(1%)	-1

Features:

```
Query  854   MKKIITVSAMAMAGMFSTQTLADEGKTGFYVTGKAGASVVTQIDQRFRQDFGDVVYKYKG  675
             MKK+I VSA+AMAG+FS Q LAD GKTGFYVTGKAGASVVTQ DQRFRQDFGD VYKYKG
Sbjct  1     MKKVIVVSALAMAGVFSAQALADRGKTGFYVTGKAGASVVTQTDQRFRQDFGDDVYKYKG  60

Query  674   SDKNDTVFGAGLAVGYDFYQHYNVPVRTEVEFTGGSDLYSAEEAEGYLQP--GGGTQGIQ  501
              DKNDTVFGAGLAVGYDFYQHYNVPVRTEVEF G     S    + +  P   GG +  Q
Sbjct  61    GDKNDTVFGAGLAVGYDFYQHYNVPVRTEVEFYGRGAADSHYTLDTWHSPMGDGGREDTQ  120

Query  500   ERI  492
              R+
Sbjct  121   NRL  123
```

adhesin, partial [Escherichia coli]
Sequence ID: **ref|WP_059327261.1|** Length: 225 Number of Matches: 1

See 1 more title(s)
Range 1: 1 to 123

Score	Expect	Method	Identities	Positives	Gaps	Frame
175 bits(443)	2e-49()	Compositional matrix adjust.	89/123(72%)	96/123(78%)	2/123(1%)	-1

Features:

```
Query  854   MKKIITVSAMAMAGMFSTQTLADEGKTGFYVTGKAGASVVTQIDQRFRQDFGDVVYKYKG  675
             MKK+I VSA+AMAG+FS Q LAD GKTGFYVTGKAGASVVTQ DQRFRQDFGD VYKYKG
Sbjct  1     MKKVIVVSALAMAGVFSAQALADRGKTGFYVTGKAGASVVTQTDQRFRQDFGDDVYKYKG  60

Query  674   SDKNDTVFGAGLAVGYDFYQHYNVPVRTEVEFTGGSDLYSAEEAEGYLQP--GGGTQGIQ  501
              DKNDTVFGAGLAVGYDFYQHYNVPVRTEVEF G     S    + +  P   GG +  Q
Sbjct  61    GDKNDTVFGAGLAVGYDFYQHYNVPVRTEVEFYGRGAADSHYTLDTWHSPMGDGGREDTQ  120

Query  500   ERI  492
              R+
Sbjct  121   NRL  123
```

adhesin, partial [Escherichia coli]
Sequence ID: **ref|WP_042098513.1|** Length: 214 Number of Matches: 1
Range 1: 1 to 123

Score	Expect	Method	Identities	Positives	Gaps	Frame
174 bits(442)	2e-49()	Compositional matrix adjust.	89/123(72%)	96/123(78%)	2/123(1%)	-1

Features:

```
Query  854   MKKIITVSAMAMAGMFSTQTLADEGKTGFYVTGKAGASVVTQIDQRFRQDFGDVVYKYKG  675
             MKK+I VSA+AMAG+FS Q LAD GKTGFYVTGKAGASVVTQ DQRFRQDFGD VYKYKG
Sbjct  1     MKKVIVVSALAMAGVFSAQALADRGKTGFYVTGKAGASVVTQTDQRFRQDFGDDVYKYKG  60

Query  674   SDKNDTVFGAGLAVGYDFYQHYNVPVRTEVEFTGGSDLYSAEEAEGYLQP--GGGTQGIQ  501
              DKNDTVFGAGLAVGYDFYQHYNVPVRTEVEF G     S    + +  P   GG +  Q
Sbjct  61    GDKNDTVFGAGLAVGYDFYQHYNVPVRTEVEFYGRGAADSHYTLDTWHSPMGDGGREDTQ  120

Query  500   ERI  492
              R+
Sbjct  121   NRL  123
```

adhesin [Escherichia coli]
Sequence ID: **ref|WP_059333072.1|** Length: 232 Number of Matches: 1

See 1 more title(s)
Range 1: 1 to 123

Score	Expect	Method	Identities	Positives	Gaps	Frame
175 bits(443)	3e-49()	Compositional matrix adjust.	89/123(72%)	96/123(78%)	2/123(1%)	-1

Features:

```
Query  854   MKKIITVSAMAMAGMFSTQTLADEGKTGFYVTGKAGASVVTQIDQRFRQDFGDVVYKYKG  675
             MKK+I VSA+AMAG+FS Q LAD GKTGFYVTGKAGASVVTQ DQRFRQDFGD VYKYKG
Sbjct  1     MKKVIVVSALAMAGVFSAQALADRGKTGFYVTGKAGASVVTQTDQRFRQDFGDDVYKYKG  60

Query  674   SDKNDTVFGAGLAVGYDFYQHYNVPVRTEVEFTGGSDLYSAEEAEGYLQP--GGGTQGIQ  501
              DKNDTVFGAGLAVGYDFYQHYNVPVRTEVEF G     S    + +  P   GG +  Q
```

```
Sbjct  61   GDKNDTVFGAGLAVGYDFYQHYNVPVRTEVEFYGRGAADSHYTLDTWHSPMGDGGREDTQ  120

Query  500  ERI  492
             R+
Sbjct  121  NRL  123
```

tia invasion determinant domain protein [Escherichia coli]

Sequence ID: **ref|WP_001438278.1|** Length: 185 Number of Matches: 1

See 2 more title(s)

Range 1: 1 to 123

Score	Expect	Method	Identities	Positives	Gaps	Frame
173 bits(439)	3e-49()	Compositional matrix adjust.	88/123(72%)	96/123(78%)	2/123(1%)	-1

Features:

```
Query  854  MKKIITVSAMAMAGMFSTQTLADEGKTGFYVTGKAGASVVTQIDQRFRQDFGDVVYKYKG  675
            MKK+I VSA+AMAG+FS Q LAD GKTGFYVTGKAGAS+VTQ DQRFRQDFGD VYKYKG
Sbjct  1    MKKVIAVSALAMAGVFSAQALADRGKTGFYVTGKAGASIVTQTDQRFRQDFGDDVYKYKG  60

Query  674  SDKNDTVFGAGLAVGYDFYQHYNVPVRTEVEFTGGSDLYSAEEAEGYLQP--GGGTQGIQ  501
             DKNDTVFGAGLAVGYDFYQHYNVPVRTEVEF G     S    + +  P   GG +  Q
Sbjct  61   GDKNDTVFGAGLAVGYDFYQHYNVPVRTEVEFYGRGAADSRYTLDTWHSPMGDGGREDTQ  120

Query  500  ERI  492
             R+
Sbjct  121  NRL  123
```

hypothetical protein G768_04025 [Escherichia coli HVH 107 (4-5860571)]

Sequence ID: **gb|EQQ66869.1|** Length: 124 Number of Matches: 1

See 3 more title(s)

Range 1: 1 to 94

Score	Expect	Method	Identities	Positives	Gaps	Frame
171 bits(433)	3e-49()	Compositional matrix adjust.	83/94(88%)	86/94(91%)	0/94(0%)	-1

Features:

```
Query  854  MKKIITVSAMAMAGMFSTQTLADEGKTGFYVTGKAGASVVTQIDQRFRQDFGDVVYKYKG  675
            MKK+I VSA+AMAG+FS Q LAD GKTGFYVTGKAGASVVTQ DQRFRQDFGD VYKYKG
Sbjct  1    MKKVIVVSALAMAGVFSAQALADRGKTGFYVTGKAGASVVTQTDQRFRQDFGDDVYKYKG  60

Query  674  SDKNDTVFGAGLAVGYDFYQHYNVPVRTEVEFTG  573
             DKNDTVFGAGLAVGYDFYQHYNVPVRTEVEF G
Sbjct  61   GDKNDTVFGAGLAVGYDFYQHYNVPVRTEVEFYG  94
```

adhesin [Escherichia coli]

Sequence ID: **ref|WP_059341043.1|** Length: 227 Number of Matches: 1

See 1 more title(s)

Range 1: 1 to 123

Score	Expect	Method	Identities	Positives	Gaps	Frame
174 bits(442)	3e-49()	Compositional matrix adjust.	89/123(72%)	96/123(78%)	2/123(1%)	-1

Features:

```
Query  854  MKKIITVSAMAMAGMFSTQTLADEGKTGFYVTGKAGASVVTQIDQRFRQDFGDVVYKYKG  675
            MKK+I VSA+AMAG+FS Q LAD GKTGFYVTGKAGASVVTQ DQRFRQDFGD VYKYKG
Sbjct  1    MKKVIVVSALAMAGVFSAQALADRGKTGFYVTGKAGASVVTQTDQRFRQDFGDDVYKYKG  60

Query  674  SDKNDTVFGAGLAVGYDFYQHYNVPVRTEVEFTGGSDLYSAEEAEGYLQP--GGGTQGIQ  501
             DKNDTVFGAGLAVGYDFYQHYNVPVRTEVEF G     S    + +  P   GG +  Q
Sbjct  61   GDKNDTVFGAGLAVGYDFYQHYNVPVRTEVEFYGRGAADSHYTLDTWHSPMGDGGREDTQ  120

Query  500  ERI  492
             R+
Sbjct  121  NRL  123
```

hypothetical protein PU18_24840, partial [Escherichia coli]

Sequence ID: **gb|KHI75040.1|** Length: 119 Number of Matches: 1

Range 1: 1 to 94

Score	Expect	Method	Identities	Positives	Gaps	Frame
171 bits(432)	3e-49()	Compositional matrix adjust.	83/94(88%)	86/94(91%)	0/94(0%)	-1

Features:

```
Query  854  MKKIITVSAMAMAGMFSTQTLADEGKTGFYVTGKAGASVVTQIDQRFRQDFGDVVYKYKG  675
            MKK+I VSA+AMAG+FS Q LAD GKTGFYVTGKAGASVVTQ DQRFRQDFGD VYKYKG
```

```
Sbjct  1    MKKVIVVSALAMAGVFSAQ̆ALADRGKTGFYVTGKAGASVVTQ̆TDQ̆RFRQ̆DFGDDVYKYKG  60

Query  674  SDKNDTVFGAGLAVGYDFYQHYNVPVRTEVEFTG  573
             DKNDTVFGAGLAVGYDFYQ̆HYNVPVRTEVEF G
Sbjct  61   GDKNDTVFGAGLAVGYDFYQ̆HYNVPVRTEVEFYG  94
```
